# Frequency of subtype B and F1 dual infection in HIV-1 positive, Brazilian men who have sex with men

**DOI:** 10.1186/1743-422X-9-223

**Published:** 2012-09-29

**Authors:** Ana Carolina Soares de Oliveira, Rodrigo Pessôa de Farias, Antonio Charlys da Costa, Mariana Melillo Sauer, Katia Cristina Bassichetto, Solange Maria Santos Oliveira, Priscilla Ramos Costa, Claudia Tomiyama, Helena Tomoko Iwashita Tomiyama, Ester Cerdeira Sabino, Esper Georges Kallas, Sabri Saeed Sanabani

**Affiliations:** 1 Department of Translational Medicine, Federal University of São Paulo, São Paulo, Brazil; 2 São Paulo Institute of Tropical Medicine, University of São Paulo, São Paulo, Brazil; 3 Division of Clinical Immunology and Allergy, School of Medicine, University of São Paulo, São Paulo, Brazil; 4 Public Health Department of São Paulo, São Paulo, Brazil; 5 Department of Infectious Diseases, School of Medicine, University of São Paulo, São Paulo, Brazil; 6 Clinical Laboratory, Department of Pathology, LIM 03, Hospital das Clínicas (HC), School of Medicine, University of São Paulo, São Paulo, Brazil; 7 Faculdade de Medicina, Instituto de Medicina Tropical de São Paulo, Universidade de São Paulo, LIM 52 - Av. Dr. Enéas Carvalho de Aguiar, 470 - 2 andar - Cerqueira Cesar, 05403-000, Sao Paulo, SP, Brasil

## Abstract

**Background:**

Because various HIV vaccination studies are in progress, it is important to understand how often inter- and intra-subtype co/superinfection occurs in different HIV-infected high-risk groups. This knowledge would aid in the development of future prevention programs. In this cross-sectional study, we report the frequency of subtype B and F1 co-infection in a clinical group of 41 recently HIV-1 infected men who have sex with men (MSM) in São Paulo, Brazil.

**Methodology:**

Proviral HIV-1 DNA was isolated from subject's peripheral blood polymorphonuclear leukocytes that were obtained at the time of enrollment. Each subject was known to be infected with a subtype B virus as determined in a previous study. A small fragment of the *integrase* gene (nucleotide 4255–4478 of HXB2) was amplified by nested polymerase chain reaction (PCR) using subclade F1 specific primers. The PCR results were further confirmed by phylogenetic analysis. Viral load (VL) data were extrapolated from the medical records of each patient.

**Results:**

For the 41 samples from MSM who were recently infected with subtype B virus, it was possible to detect subclade F1 proviral DNA in five patients, which represents a co-infection rate of 12.2%. In subjects with dual infection, the median VL was 5.3 × 10^4^ copies/ML, whereas in MSM that were infected with only subtype B virus the median VL was 3.8 × 10^4^ copies/ML (p > 0.8).

**Conclusions:**

This study indicated that subtype B and F1 co-infection occurs frequently within the HIV-positive MSM population as suggested by large number of BF1 recombinant viruses reported in Brazil. This finding will help us track the epidemic and provide support for the development of immunization strategies against the HIV.

## Introduction

Mutation and recombination are the two main evolutionary forces that generate genetic variation in HIV-1. Like other human positive-sense RNA viruses, human immunodeficiency virus (HIV-1) has a high mutation rate, which in its case is due to the error-prone nature of the viral reverse transcriptase (3 × 10^-5^ mutations per nucleotide per replication cycle) [1,2]. This high rate of mutation, coupled with a high replication rate (10.3 × 10^9^ particles per day) [3], allows for the generation and fixation of a variety of advantageous mutations in a virus population. These changes are selected in response to the host immune pressure to enable the virus to resist the host defense. Recombination is another potential source of genetic variation that contributes significantly to the genetic diversification of HIV and could potentially produce more virulent viruses, drug resistant viruses, or viruses with altered cell tropism that may reduce the effectiveness of antiretroviral therapy and may present major challenges for the design of vaccines [4]. It has been reported that recombinant viruses including the unique recombinant forms (URF) and circulating recombinant forms (CRF), may account for at least 20% of all HIV infections [5]. The existence of recombinant viruses is an evidence of simultaneous infection of multiple viruses during a single transmission event (co-infection) or from the sequential infection of viruses during multiple transmission events (superinfection). Co-infection has been well documented in individuals that are infected with both HIV-1 and HIV-2 [6,7], and individuals infected with viruses from different HIV-1 groups [8], and individuals infected with different subtypes or recombinant variants [9-16], and with divergent variants of the same subtype from different sources [17-23]. The consequence of co-infection has significant implications on antiretroviral resistance and vaccine development. Furthermore, it could lead to immunologic escape and subsequent disease progression [21,24]. Thus, determining the frequency of dual infections is of great interest for the clinical management of HIV infection.

Unpublished data from our laboratory found evidence of an HIV-1 subtype B and F1 dual infected homosexual patient. Therefore, we attempted to retrospectively evaluate the frequency of HIV-1 subclade F1 and subtype B dual infections in Brazilian recently HIV-1-infected men who have sex with men (MSM).

## Materials and methods

### Patients

The subjects in this study were part of a previously described prospective cohort of recently HIV-1 infected persons from São Paulo, Brazil [25]. Recent infection was defined as being infected for less than 170 days (95% confidence interval: 145–200 days) using the serologic testing algorithm for recent human immunodeficiency virus (HIV) seroconversion (STARHS) strategy [26]. Forty-one MSM participants were selected for this study based on the following criteria: infection with a subtype B virus based on near full-length genome or partial *pol* (including complete *integrase* region) analysis [27,28], availability of a blood sample from the initial time point. The details of this cohort and the methods for identifying recent infection were described elsewhere [25,27,29]. For this study, evidence for dual infection was defined as the presence of subclade F1-proviral DNA in the same blood samples and in the same genomic region that was previously identified for subtype B infection. Detection of both viruses in a single clinical sample strongly suggests that the two variants were due to either co-infection or superinfection. However, only the frequency of dual infection was concluded in this study because we do not know whether co-infection or superinfection originally occurred. Patient data, including age, number of CD4-positive T cells, and viral load (VL) was obtained from the patient medical records (Table 1). Information on the sexual behaviors, including the specific number of unprotected sexual acts, sexual partners, sex acts per partner, the HIV status of a partner and VL of an HIV-1 positive partner at the time of sexual intercourse are lacking. All study participants signed an informed consent form, and the project was approved by the ethics committee of the Federal University of São Paulo.

**Table 1:** Characteristics of the 41 study MSM subjects

Median age. years (range)	30.6 (18–56)
Median CD4 count . cells/mm3 (range)	564 (198–2449)
Median viral load. log HIV RNA copies/mL (range)	4.3 X 10^4^ (< 400–39 X10^4^)
Serologies (% positive)*	
HHV8 ( LANA)	11 (26.8)
TPHA positive	10 (24.3)
Toxoplasmosis [30]	26 (63.4)
Anti-HCV	0
Anti-HBc (%)	18 (43.9)

* *HHV8* Human herpes virus type 8; *LANA* latency-associated nuclear antigen; *TPHA Treponema pallidum* hemagglutination assay; *HCV* Hepatitis C virus; *HBc* Hepatitis B core antigen.

### Proviral DNA extraction and amplification

All samples used in this study were the same as those described previously [27]. PMNs were isolated from patient blood samples collected at the time of enrollment, and genomic DNA was extracted using a QIAamp DNA Blood Mini Kit (Qiagen, Hilden, Germany), according to the manufacturer’s instructions. The proviral DNA was subjected to nested PCR to amplify a 248 bp fragment of the *integrase* gene (*pol*-IN) using the primers listed in Table 2. The assay was developed to detect subclade F1 viral isolates with a high sensitivity and specificity. By analyzing the alignment of complete genome sequences of different subtypes of HIV-1, the *pol*-IN gene region was targeted because it was well conserved within subclade F1 strains, yet has a unique sequence compared to other HIV-1 subtypes. In the initial PCR, a 266 bp *integrase* fragment was amplified using an Eppendorf Master cycler. The PCR condition consisted of an initial denaturation step at 95°C for 2 minutes, followed by 35 cycles of 95°C for 30 seconds, 58°C for 30 seconds and 68°C for 1 minute, and a final extension was carried out at 68°C for 5 minutes. For the nested PCR, 5 μl of the first PCR reaction was used, and the PCR mix included subclade F1-specific inner primers (Table 2). The amplified product was electrophoresed through a 1.5% (wt/vol) agarose gels containing 0.5 × Tris Borate EDTA followed by ethidium bromide staining. To allow a more advanced phylogenetic analysis, another set of forward primers that were specific for subclade F1 *pol* region were designed. These primers were used in combination with the reverse *pol*-IN primers to amplify a 1247 bp L-*pol* fragment. Amplification and detection of the L-*pol* fragment was carried out using the same PCR conditions as described previously with a modification of the extension time to 2 minutes.

**Table 2:** Details of PCR primer combinations

**N0.**	**Primer**	**Combinations of Primers**	**Fragment amplified**	**PCR type**	**sequence (5’-3’)**	**Primer Position**^**3**^	**Product size bp**
1	DPSH-O-Fwd^1^	1+2	*pol*-IN	Outer	ATAAGGCACAGGAGGAACATGAAAAATATCACAAC	4246-4280	266
2	DPLINF-O-Rev^2^				TTTTTTTTTCTGCTGGGATAACTTCTGCTTCTAGG	4512-4478	
3	DPSH-N-Fwd	3+4		Nested	AGGAGGAACATGAAAAATATCACAAC	4255-4280	248
4	DPINF-N-Rev				CTGCTGGGATAACTTCTGCTTCTAGG	4503-4478	
5	DPINF-O-Fwd	5+2	L-*pol*	Outer	TTTTTTTTTTTCTGATAAATGGACAGTGCAGCCTATACAAT	3247-3285	1265
6	DPINF-N-Fwd	6+4		Nested	CTGATAAATGGACAGTGCAGCCTATACAAT	3256-3285	1247
7	B01-Fwd	7+2	M-*pol*	Outer	TGGGTTATGAACTCCATCCTG	3238-3258	1274
8	B02-Fwd	8+4		Nested	CTGGATTCCTGAGTGGGAGTT	3776-3796	727

The underlined nucleotides (non-HIV-1 specific sequences) are tails at the 5’ end of the outer reverse primers and were added to enhance the nested amplification with inner reverse primers.

^1^ Fwd: Forward, ^2^ Rev: Reverse.

^3^ Nucleotide position of the primer according to the HXB2 sequence (K03455) numbering.

Isolates that were characterized as subclade F1 by PCR amplification and DNA sequencing of the *pol*-IN fragment but failed to be amplified by PCR using the L-*pol* specific primers suggested that these isolates may be recombinant viruses. To address this issue, forward primers were used to amplify a 727-bp product (denoted as M-*pol*). These primers were able to amplify a broad range of HIV-1 variants including subtype B and F1. These primers were used in combination with the reverse *pol*-IN primers in a nested PCR assay, using the same conditions described for the *pol*-IN PCR assay except for an annealing temperature at 55°C and a 2 minute extension time. Both PCR assays, L-*pol* and M-*pol*, had a sensitivity of 25 and 15 copies per reaction, respectively. All assays were performed in duplicate for each fragment using the primer combinations shown in Table 2. Positive and negative controls (healthy donor PMNs) were included in each assay. Strict laboratory precautions were taken to avoid cross contamination. Specimens that had a clear amplification in each duplicate reaction were considered to be positive.

### Sequencing and phylogenetic analysis

The amplified DNA fragments were purified using a QIAquick PCR Purification Kit (Qiagen, Hilden, Germany) and directly sequenced using second-round primers and the PRISM Big Dye Terminator Cycle Sequencing Ready Reaction Kit (Applied Biosystems/Perkin-Elmer, Foster City, CA) on an automated sequencer (ABI 3130, Applied Biosystems). After excluding the primer regions, each amplicon was assembled into a contiguous sequence alignment and edited with the Sequencher program 4.7 (Gene Code Corp., Ann Arbor, MI). The alignment of multiple sequences, including the reference sequences for subtypes A–D, F–H, J and K ( http://hiv-web.lanl.gov), was performed using the CLUSTAL X program [30] and followed by manual editing using the BioEdit Sequence Alignment Editor program version 5.0.7 [31]. Gaps and ambiguous positions were removed from the final alignment. The phylogenetic relationships were determined by two methods: the neighbor-joining (NJ) algorithm of MEGA version 5.0 software [32] and maximum likelihood (ML) using PHYML v.2.4.4 [33]. For the NJ method, trees were constructed under the maximum composite likelihood substitution model and bootstrap re-sampling was carried out 1000 times. For the ML method, phylogentic trees were constructed using the GTR + I + G substitution model and a BIONJ starting tree. Heuristic tree searches under the ML optimality criterions were performed using the nearest-neighbor interchange (NNI) branch-swapping algorithm. The approximate likelihood ratio test (aLRT) based on a Shimodaira-Hasegawa-like procedure was used as a statistical test to calculate branch support. Trees were displayed using the MEGA v.5 package.

### Nucleotide sequence accession numbers

The sequences described here have been deposited (accession numbers pending)

## Results

**Figure 1:**
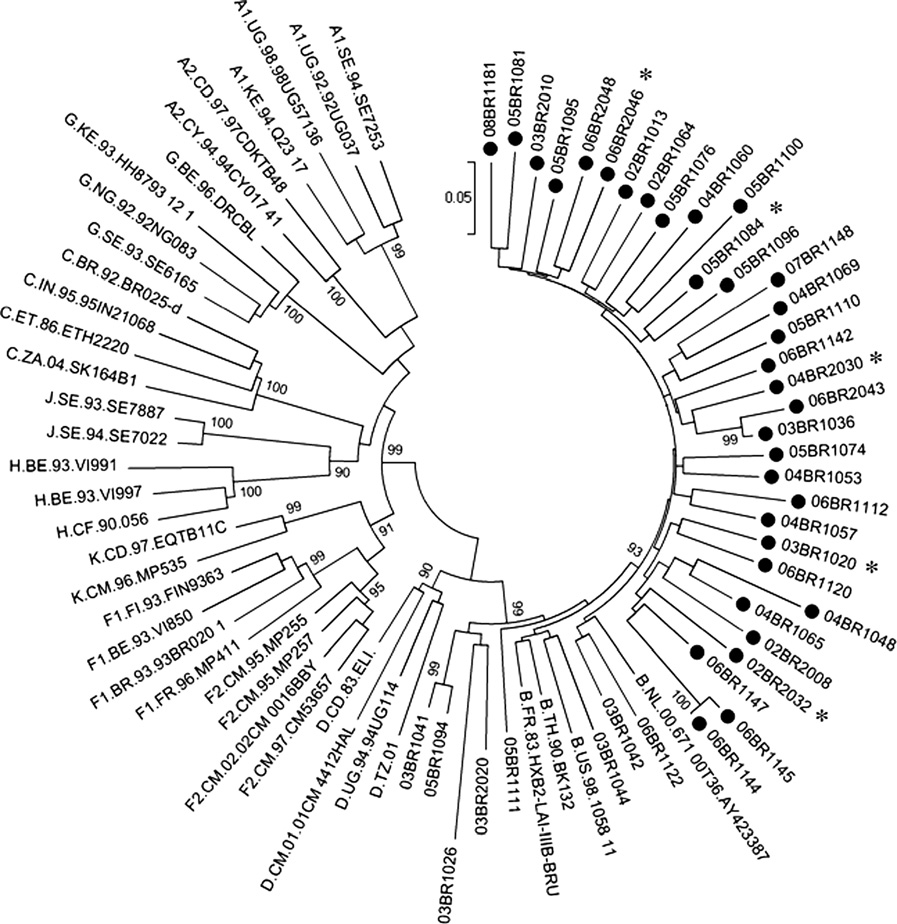
**Phylogenetic tree constructed using a maximum-likelihood method from partial *pol *region (1279 bp; nt 3822–5101 of HXB2) of 41 samples from MSM that have previously been determined to be infected with HIV-1 subtype B (indicated by black circles) and 37 HIV-1 reference sequences from the Los Alamos HIV-1 database representing 11 genetic subtypes.** Samples that were identified in this study to host subclade F1 DNA are indicated with star symbol. For purposes of clarity, the tree was midpoint rooted. The approximate likelihood ratio test (aLRT) values of ≥ 90% are indicated at nodes. The scale bar represents 0.05 nucleotide substitutions per site.

Blood samples were obtained from 41 MSM study participants who had been previously characterized as being infected with HIV-1 subtype B virus (Figure 1) [27]. The median VL in this cohort was 4.3 × 10^4^ copies/ml (range, <400-39.3 × 10^4^). The median baseline CD4-positive T cell count was 564 cells/mm^3^ (range, 198–2449 cells/mm^3^). The age of the subjects ranged from 18 to 56 years, and the median age was 30.6 years. All patients were treatment-naive at the time of sample collection. The main characteristics of the study population are shown in Table 1.

**Figure 2:**
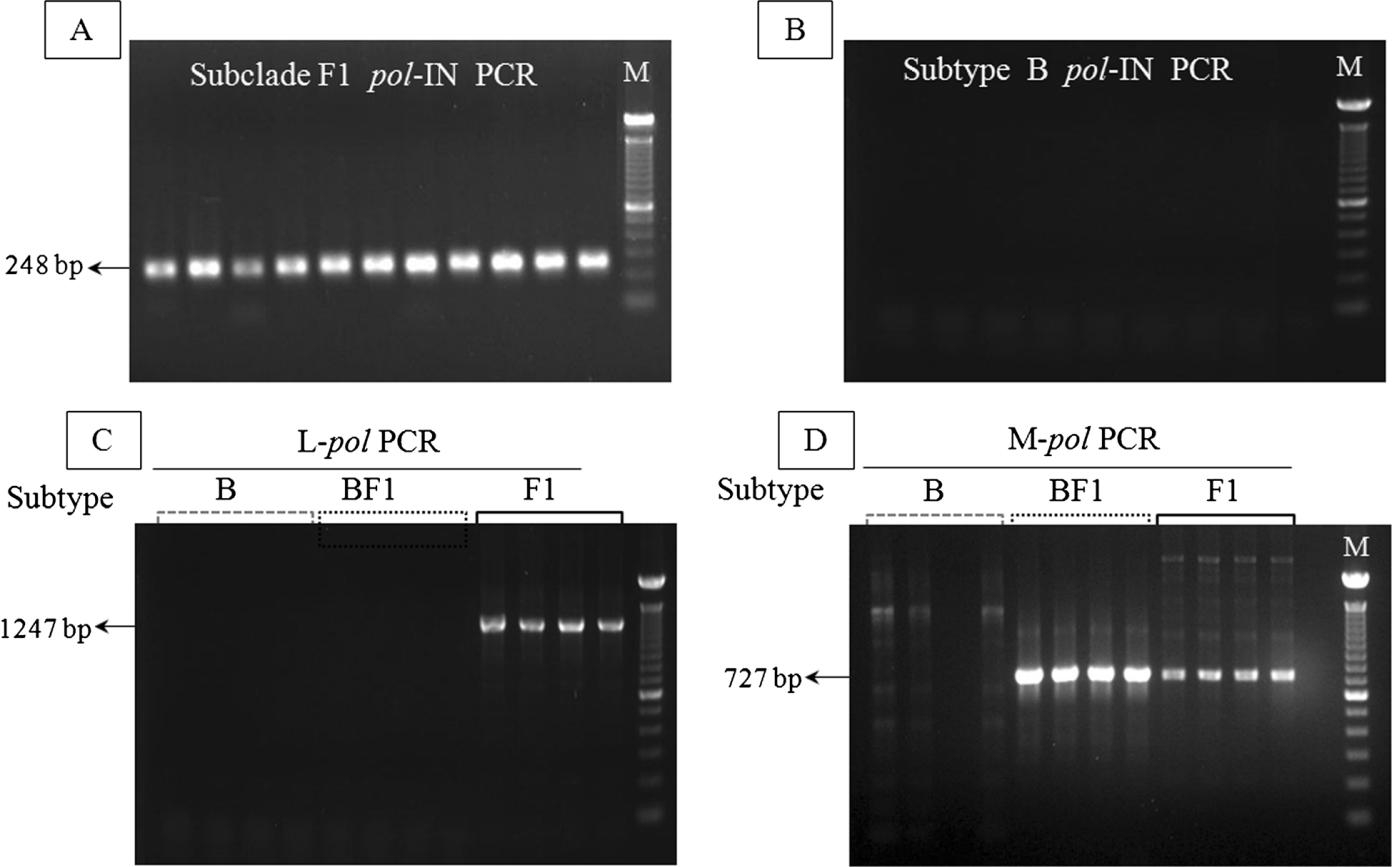
**Representative agarose gel of the nested polymerase chain reaction (PCR) produts.** Electrophoresis and subsequent ethidium bromide staining of amplicons from the nested PCR amplification with *pol*-IN subclade F1-specific primers of the DNA samples known to harbor HIV-1 subclade F1 (**A**), and subtype B (**B**). Amplicons of nested PCR (1247 bp) performed with L-*pol* subclade F1-specific primers using samples that were observed to be infected with HIV-1 subclade F1 (solid line), BF1 recombinants with breakpoints located between the PCR primers (dotted line), and subtype B (dashed line). (**C**). Amplicons of nested PCR (727 bp) performed with M-*pol* primers (specific for F1 and BF1 variants) using samples observed to be infected with HIV-1 subclade F1 (solid line), BF1 recombinants with breakpoints located between the PCR primers (dotted line), and subtype B (dashed line) (**D**). M, molecular weight DNA marker (100 bp DNA ladder, Invitrogen).

Before processing the patient samples, we wanted to determine the sensitivity of the subclade F1 specific primers for the amplification of the *pol*-IN fragment. This was performed by using multiple reaction tubes, each containing 10^5^ copies of HIV-1 subtype B and a known quantity of subclade F1 partial proviral *pol* DNA ranging from 1 × 10^0^ to 1 × 10^6^ copies per reaction. The sensitivity of PCR amplification of the subclade F1 partial proviral *pol* DNA was one copy of target per reaction in a background of 10^5^ copies of HIV-1 subtype B. The specificity of the nested PCR for subclade F1 was confirmed by sequencing the amplified PCR products. Furthermore, this assay was tested on 15 subclade F1 and 25 subtype B patient samples that were previously characterized by partial and near full-length proviral genome analysis [27,28,34,35]. As shown in Figure 2A, clear bands at the expected size of 248 bp for subclade F1 were seen in all reactions, but not in the subtype B strains (Figure 2B).

Having verified the sensitivity and specificity of the *pol*-IN primers, we wanted to establish the conditions and reliability of the L-*pol* and M-*pol* primers in amplifying their specific PCR fragments (see Table 2). Thus, the latter primers were tested against a range of previously published HIV-1 subtype B, F1 and BF1 proviral variants [27,28,34-37]. The L-*pol* primers amplified a clear product from only subclade F1 isolates (Figure 2C). Similarly, the M-*pol* primers amplified a fragment of the correct size from all BF1 and subclade F1 variants, but no or weak amplification of multiple products was observed when the primers were used to assay subtype B variants (Figure 2D). All products were sequenced to determine the specific subtype.

**Figure 3:**
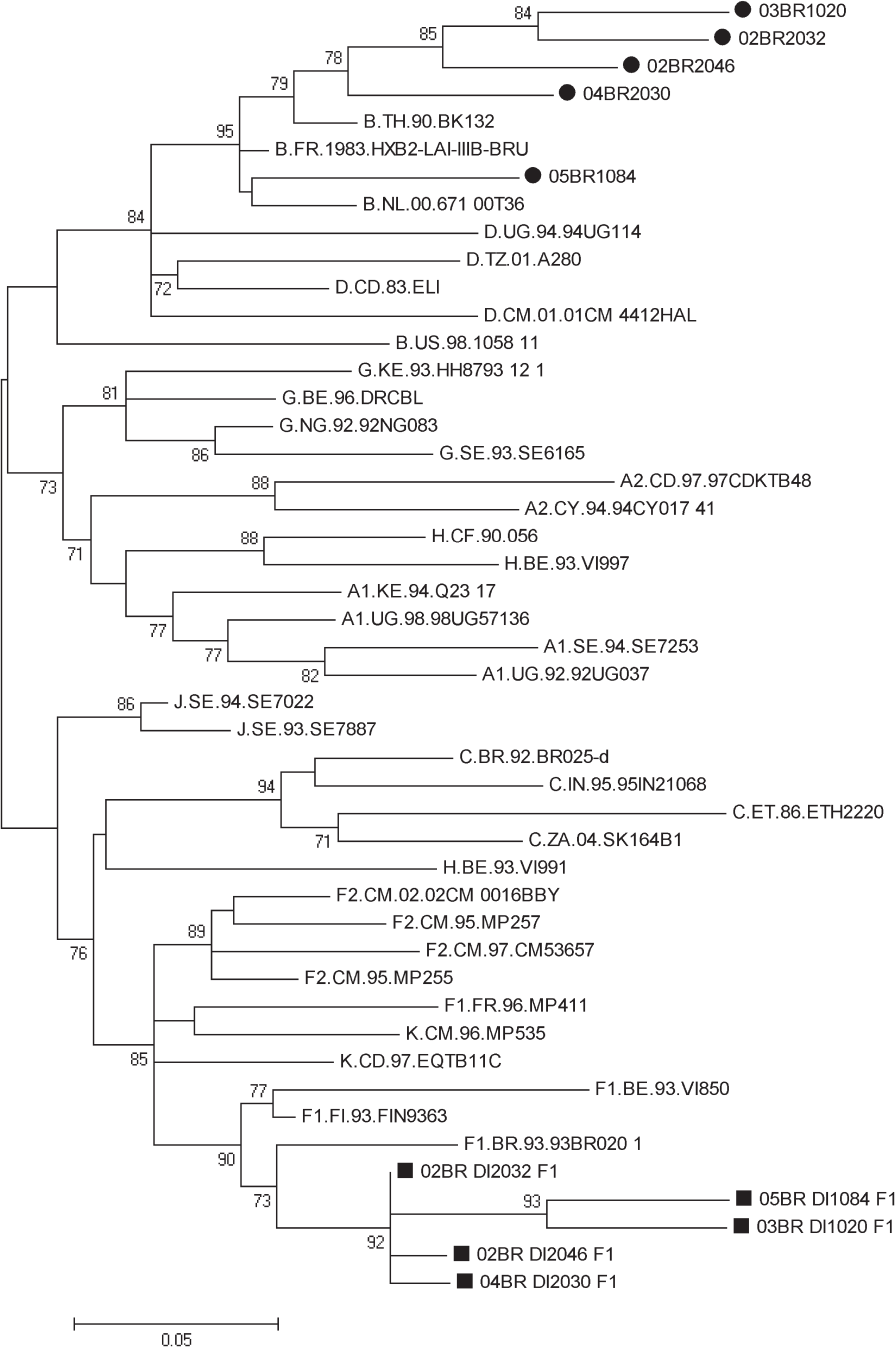
**Phylogenetic tree constructed using a maximum-likelihood method from *pol*-IN (219 bp ; nt 4269–4488 of HXB2) fragments from five of the samples isolated from MSM observed to be infected with both subtype B (indicated by black circles) and subclade F1 DNA (indicated by black squares) along with HIV-1 reference sequences from the Los Alamos HIV-1 database representing 11 genetic subtypes.** For purposes of clarity, the tree was midpoint rooted. The approximate likelihood ratio test (aLRT) values of ≥ 70% are indicated at nodes. The scale bar represents 0.05 nucleotide substitutions per site.

Using *pol*-IN nested PCR in our clinical samples, 5 of 41 (12.2%) of the MSM patient samples, already described to be infected with subtype B, were also found to be infected with F1 virus. The sequences of the *pol*-IN fragment from the five subjects were then compared with representative sequences from all subtypes available in the HIV database (year 2005). Consistent with the results of our *pol*-IN PCR assay, the ML tree, shown in Figure 3, indicated that all five sequences clustered together with the subclade F1 (90% aLRT) reference strain. The mean intersubject sequence diversity among these five isolates was 1.1% (range, 0.2–1.7%). As shown in Figure 1, HIV-1subtype B strains from these 5 MSM did not demonstrate any evolutionary linkages.

Despite various attempts, amplification of the L-*pol* fragment in subclade F1 positive samples was unsuccessful. These results suggested that the 5 MSM were co-infected with a BF1 recombinant virus. To address this question, proviral DNA from the samples that were positive for the *pol*-IN fragment were subjected to amplification and sequencing of M-*pol*. We used a combination of primers that specifically amplified the M-*pol* fragment that contained subclade F1 sequence at the 3' end if these patients were infected with recombinant virus. This reaction produced either no amplification or resulted in the amplification of multiple weak fragments with insufficient yield to perform sequencing. Overall, these results indicated that the 5 MSM patients are likely infected with both subtype B and F1 HIV-1. The failure to amplify L-*pol* and M-*pol* fragments is likely a result of the low frequency of subclade F1 proviral DNA.

A comparison of the VLs between single and dual infected MSM was performed. In subjects with dual infection, the median VL was 5.3 × 10^4^ and ranged from 1.5 × 10^4^ to 12.5 × 10^4^ copies/mL. In MSM that were infected only with subtype B, the median VL was 3.8 × 10^4^ copies/mL and ranged from undetectable (<400 copies/mL) to 39.3 × 10^4^ copies/mL. As observed in other studies, there results indicated that the VL are statistically the same [38].

## Discussion

This study describes the prevalence of HIV-1 subtype B and F1 dual infections in recently infected Brazilian MSM. Among the 41 subjects studied, 12.2% were positive for both subtypes B (from previous study) and F1 proviral DNA (current study). These results are not surprising because both viral subtypes and recombinants are widely circulating in Brazil, which is a country that offers an excellent setting for such studies. It is probable that our results have underestimated the true rate of dual infection in this group. The most likely explanation for underestimation is that some isolates could have been undetected by our specific PCR screening method because of a mismatch at the primer binding sites, low proviral load, or that the subclade F1 isolates were maintained in another reservoir other than the CD4-positive compartment that was sampled in the peripheral blood. Additionally, our method only detects dual infection of subtypes B and F1 when the *pol*-IN region is subclade F1. We could have missed some instances of co-infection if recombination had happened, and the *pol*-IN fragment is not subclade F1. Therefore, it is possible that the dual infection in this group may be higher than what was observed if we had sequenced a larger region or sequenced other regions of the viral genome. Our attempts to amplify larger fragments to determine if recombination had occurred were unsuccessful, probably because these subjects were co-infected and maintained low proviral loads of subclade F1 compared to the subtype B viral population. However, because most of the HIV-1 subclade F1 strains circulating in Brazil contain recombinant genomes and particularly with recombination with subtype B [27,34,39], we cannot rule out the possibility of F1 recombination in the present study. Our findings also may not be generalizable. This retrospective study focused on a select, relatively small group of recently HIV infected MSM that were known to be infected with subtype B virus. Consequently, the dual infection rates reported cannot necessarily be extrapolated to other populations of HIV-infected MSM. In spite of these caveats, we have been able to identify five cases of dual infection by studying only one genomic region of the HIV genome, similar to the Kenyan study [38]. In the Kenyan study, the authors observed seven cases of HIV-1 superinfection among 36 high-risk women and only five cases of superinfection were detected by differences in only one gene. Unexpectedly, a lack of detectable HIV-1 dual infections has recently been reported in a retrospective study of 83 samples from chronically infected patients on antiretroviral treatment throughout the KwaZulu-Natal region that has a high HIV prevalence [40]. The lack of dual infections in this study was explained by the ability of the immune system to evolve overtime to eliminate or prevent a second viral infection during chronic infection [40,41].

Despite a large body of literature, the true prevalence and the timing of immune selection in HIV co/superinfection cases have not yet been substantiated by robust clinical studies and the limited data that do exist have yielded inconclusive or contradictory findings that partially contribute to the controversies surrounding the challenge and implications of HIV co/superinfection for efficient vaccine design [38,42,43]. Our estimates of subtype B and F1 dual infection rates are not directly comparable to other published studies as each study group has used different research designs, methodologic approaches, and different target population for the search of different HIV-1 subtypes [8,16,44-52]. All together, these data lend further support to the conclusions that dual infections are an integral part of the HIV/AIDS epidemic, particularly in countries where multiple subtypes are circulating in the population [15].

In summary, our data adds to the knowledge of the prevalence of HIV-1 dual infections caused by HIV-1 subtype B and F1 viruses in MSM subjects and provides data from a country where such a phenomenon is rarely documented. Furthermore, these data agree with the consensus that the presence of two or more HIV-1 subtypes within an infected individual is relatively frequent [53,54]. Thus, testing for co-infection and superinfection and the implementation of effective preventative measures in the MSM population remains relevant issue.

## Competing interests

The authors declare that they have no competing interests.

## Authors’ contributions

ACSO, RPF, ACC and SSS conceived and designed the study, performed the experiments analyzed the data and wrote the paper. MMS collected clinical data, KCB, SMSO, PRC, CT, and HTIT contributed reagents/materials/analysis tools. ECS EGK and SSS critically reviewed the paper and secured funding. All authors read and approved the final manuscript.

## References

[B1] Mansky LM The mutation rate of human immunodeficiency virus type 1 is influenced by the vpr gene Virology 1996 222 2 391 400 10.1006/viro.1996.0436 8806523

[B2] Mansky LM Temin HM Lower in vivo mutation rate of human immunodeficiency virus type 1 than that predicted from the fidelity of purified reverse transcriptase J Virol 1995 69 8 5087 5094 7541846 10.1128/jvi.69.8.5087-5094.1995 PMC189326

[B3] Perelson AS Neumann AU Markowitz M Leonard JM Ho DD HIV-1 dynamics in vivo: virion clearance rate, infected cell life-span, and viral generation time Science 1996 271 5255 1582 1586 10.1126/science.271.5255.1582 8599114

[B4] Cohen OJ Fauci AS Transmission of multidrug-resistant human immunodeficiency virus–the wake-up call N Engl J Med 1998 339 5 341 343 10.1056/NEJM199807303390511 9682050

[B5] Arien KK Vanham G Arts EJ Is HIV-1 evolving to a less virulent form in humans? Nat Rev Microbiol 2007 5 2 141 151 10.1038/nrmicro1594 17203103 PMC7097722

[B6] Wiktor SZ Nkengasong JN Ekpini ER Adjorlolo-Johnson GT Ghys PD Brattegaard K Tossou O Dondero TJ De Cock KM Greenberg AE Lack of protection against HIV-1 infection among women with HIV-2 infection AIDS 1999 13 6 695 699 10.1097/00002030-199904160-00010 10397564

[B7] Gunthard HF Huber M Kuster H Shah C Schupbach J Trkola A Boni J HIV-1 superinfection in an HIV-2-infected woman with subsequent control of HIV-1 plasma viremia Clin Infect Dis 2009 48 11 e117 e120 10.1086/598987 19382874

[B8] Takehisa J Zekeng L Miura T Ido E Yamashita M Mboudjeka I Gurtler LG Hayami M Kaptue L Triple HIV-1 infection with group O and Group M of different clades in a single Cameroonian AIDS patient J Acquir Immune Defic Syndr Hum Retrovirol 1997 14 1 81 82 10.1097/00042560-199701010-00015 8989217

[B9] Thomson MM Delgado E Manjon N Ocampo A Villahermosa ML Marino A Herrero I Cuevas MT Vazquez-de Parga E Perez-Alvarez L HIV-1 genetic diversity in Galicia Spain: BG intersubtype recombinant viruses circulating among injecting drug users AIDS 2001 15 4 509 516 10.1097/00002030-200103090-00010 11242148

[B10] Hoelscher M Dowling WE Sanders-Buell E Carr JK Harris ME Thomschke A Robb ML Birx DL McCutchan FE Detection of HIV-1 subtypes, recombinants, and dual infections in east Africa by a multi-region hybridization assay AIDS 2002 16 15 2055 2064 10.1097/00002030-200210180-00011 12370505

[B11] Iversen AK Learn GH Fugger L Gerstoft J Mullins JI Skinhoj P Presence of multiple HIV subtypes and a high frequency of subtype chimeric viruses in heterosexually infected women J Acquir Immune Defic Syndr 1999 22 4 325 332 10634193 10.1097/00126334-199912010-00002

[B12] Artenstein AW VanCott TC Mascola JR Carr JK Hegerich PA Gaywee J Sanders-Buell E Robb ML Dayhoff DE Thitivichianlert S Dual infection with human immunodeficiency virus type 1 of distinct envelope subtypes in humans J Infect Dis 1995 171 4 805 810 10.1093/infdis/171.4.805 7706806

[B13] Becker-Pergola G Mellquist JL Guay L Mmiro F Ndugwa C Kataaha P Jackson JB Eshleman SH Identification of diverse HIV type 1 subtypes and dual HIV type 1 infection in pregnant Ugandan women AIDS Res Hum Retroviruses 2000 16 12 1099 1104 10.1089/088922200414938 10954884

[B14] Janini LM Pieniazek D Peralta JM Schechter M Tanuri A Vicente AC dela Torre N Pieniazek NJ Luo CC Kalish ML Identification of single and dual infections with distinct subtypes of human immunodeficiency virus type 1 by using restriction fragment length polymorphism analysis Virus Genes 1996 13 1 69 81 10.1007/BF00576981 8938982

[B15] Ramos A Tanuri A Schechter M Rayfield MA Hu DJ Cabral MC Bandea CI Baggs J Pieniazek D Dual and recombinant infections: an integral part of the HIV-1 epidemic in Brazil Emerg Infect Dis 1999 5 1 65 74 10.3201/eid0501.990108 10081673 PMC2627691

[B16] Andreani G Espada C Ceballos A Ambrosioni J Petroni A Pugliese D Bouzas MB Fernandez Giuliano S Weissenbacher MC Losso M Detection of HIV-1 dual infections in highly exposed treated patients Virol J 2011 8 392 10.1186/1743-422X-8-392 21824422 PMC3163559

[B17] Diaz RS Sabino EC Mayer A Mosley JW Busch MP Dual human immunodeficiency virus type 1 infection and recombination in a dually exposed transfusion recipient. The Transfusion Safety Study Group J Virol 1995 69 6 3273 3281 7745674 10.1128/jvi.69.6.3273-3281.1995 PMC189038

[B18] Liu SL Mittler JE Nickle DC Mulvania TM Shriner D Rodrigo AG Kosloff B He X Corey L Mullins JI Selection for human immunodeficiency virus type 1 recombinants in a patient with rapid progression to AIDS J Virol 2002 76 21 10674 10684 10.1128/JVI.76.21.10674-10684.2002 12368309 PMC136598

[B19] Zhu T Wang N Carr A Wolinsky S Ho DD Evidence for coinfection by multiple strains of human immunodeficiency virus type 1 subtype B in an acute seroconvertor J Virol 1995 69 2 1324 1327 7815515 10.1128/jvi.69.2.1324-1327.1995 PMC188714

[B20] Sala M Pelletier E Wain-Hobson S HIV-1 gp120 sequences from a doubly infected drug user AIDS Res Hum Retroviruses 1995 11 5 653 655 10.1089/aid.1995.11.653 7576923

[B21] Gottlieb GS Nickle DC Jensen MA Wong KG Grobler J Li F Liu SL Rademeyer C Learn GH Karim SS Dual HIV-1 infection associated with rapid disease progression Lancet 2004 363 9409 619 622 10.1016/S0140-6736(04)15596-7 14987889

[B22] Ssemwanga D Ndembi N Lyagoba F Bukenya J Seeley J Vandepitte J Grosskurth H Kaleebu P HIV type 1 subtype distribution, multiple infections, sexual networks, and partnership histories in female sex workers in Kampala, Uganda AIDS Res Hum Retroviruses 2012 28 4 357 365 10.1089/aid.2011.0024 21749285

[B23] Vandepitte J Bukenya J Weiss HA Nakubulwa S Francis SC Hughes P Hayes R Grosskurth H HIV and Other Sexually Transmitted Infections in a Cohort of Women Involved in High-Risk Sexual Behavior in Kampala, Uganda Sex Transm Dis 2011 38 4 316 323 310.1097/OLQ.1090b1013e3182099545 PMC3920055 23330152

[B24] Sagar M Lavreys L Baeten JM Richardson BA Mandaliya K Chohan BH Kreiss JK Overbaugh J Infection with multiple human immunodeficiency virus type 1 variants is associated with faster disease progression J Virol 2003 77 23 12921 12926 10.1128/JVI.77.23.12921-12926.2003 14610215 PMC262567

[B25] Kallas EG Bassichetto KC Oliveira SM Goldenberg I Bortoloto R Moreno DM Kanashiro C Chaves MM Sucupira MC Diniz A Establishment of the serologic testing algorithm for recent human immunodeficiency virus (HIV) seroconversion (STARHS) strategy in the city of Sao Paulo, Brazil Braz J Infect Dis 2004 8 6 399 406 15880230 10.1590/s1413-86702004000600003

[B26] Kothe D Byers RH Caudill SP Satten GA Janssen RS Hannon WH Mei JV Performance characteristics of a new less sensitive HIV-1 enzyme immunoassay for use in estimating HIV seroincidence J Acquir Immune Defic Syndr 2003 33 5 625 634 10.1097/00126334-200308150-00012 12902808

[B27] Sanabani SS Pastena ER da Costa AC Martinez VP Kleine-Neto W de Oliveira AC Sauer MM Bassichetto KC Oliveira SM Tomiyama HT Characterization of partial and near full-length genomes of HIV-1 strains sampled from recently infected individuals in Sao Paulo, Brazil PLoS One 2011 6 10 e25869 10.1371/journal.pone.0025869 22022460 PMC3193532

[B28] Diaz RS Leal E Sanabani S Sucupira MC Tanuri A Sabino EC Janini LM Selective regimes and evolutionary rates of HIV-1 subtype B V3 variants in the Brazilian epidemic Virology 2008 381 2 184 193 10.1016/j.virol.2008.08.014 18809195

[B29] Batista MD Ferreira S Sauer MM Tomiyama H Giret MT Pannuti CS Diaz RS Sabino EC Kallas EG High human herpesvirus 8 (HHV-8) prevalence, clinical correlates and high incidence among recently HIV-1-infected subjects in Sao Paulo Brazil. PLoS One 2009 4 5 e5613 10.1371/journal.pone.0005613 PMC2682704 19479040

[B30] Thompson JD Gibson TJ Plewniak F Jeanmougin F Higgins DG The CLUSTAL_X windows interface: flexible strategies for multiple sequence alignment aided by quality analysis tools Nucleic Acids Res 1997 25 24 4876 4882 10.1093/nar/25.24.4876 9396791 PMC147148

[B31] Hall TA BioEdit: a user-friendly biological sequence alignment editor and analysis program for windows 95/98/NT Nucleic Acids SympSer 1999 41 95 98

[B32] Tamura K Peterson D Peterson N Stecher G Nei M Kumar S MEGA5: molecular evolutionary genetics analysis using maximum likelihood, evolutionary distance, and maximum parsimony methods Mol Biol Evol 2011 28 10 2731 2739 10.1093/molbev/msr121 21546353 PMC3203626

[B33] Anisimova M Gascuel O Approximate likelihood-ratio test for branches: A fast, accurate, and powerful alternative Syst Biol 2006 55 4 539 552 10.1080/10635150600755453 16785212

[B34] Sanabani S Kleine Neto W Kalmar EM Diaz RS Janini LM Sabino EC Analysis of the near full length genomes of HIV-1 subtypes B, F and BF recombinant from a cohort of 14 patients in Sao Paulo, Brazil Infect Genet Evol 2006 6 5 368 377 10.1016/j.meegid.2006.01.003 16522378

[B35] Sanabani SS Pastena ER Kleine Neto W Barreto CC Ferrari KT Kalmar EM Ferreira S Sabino EC Near full-length genome analysis of low prevalent human immunodeficiency virus type 1 subclade F1 in Sao Paulo, Brazil Virol J 2009 6 78 10.1186/1743-422X-6-78 19531216 PMC2704198

[B36] Bimber BN Dudley DM Lauck M Becker EA Chin EN Lank SM Grunenwald HL Caruccio NC Maffitt M Wilson NA Whole-genome characterization of human and simian immunodeficiency virus intrahost diversity by ultradeep pyrosequencing J Virol 2010 84 22 12087 12092 10.1128/JVI.01378-10 20844037 PMC2977871

[B37] Tarosso LF Sauer MM Sanabani S Giret MT Tomiyama HI Sidney J Piaskowski SM Diaz RS Sabino EC Sette A Unexpected diversity of cellular immune responses against Nef and Vif in HIV-1-infected patients who spontaneously control viral replication PLoS One 2010 5 7 e11436 10.1371/journal.pone.0011436 20625436 PMC2896403

[B38] Piantadosi A Chohan B Chohan V McClelland RS Overbaugh J Chronic HIV-1 infection frequently fails to protect against superinfection PLoS Pathog 2007 3 11 e177 10.1371/journal.ppat.0030177 18020705 PMC2077901

[B39] Carr JK Avila M Gomez Carrillo M Salomon H Hierholzer J Watanaveeradej V Pando MA Negrete M Russell KL Sanchez J Diverse BF recombinants have spread widely since the introduction of HIV-1 into South America AIDS 2001 15 15 F41 47 10.1097/00002030-200110190-00002 11600844

[B40] Naidoo AF Parboosing R Gordon ML Dual HIV Infection Uncommon in Patients on Antiretroviral Therapy in a Region with High HIV Prevalence AIDS Res Hum Retroviruses 2009 25 12 1225 1230 10.1089/aid.2009.0095 20001312

[B41] Cheonis N Dual HIV infection BETA 2006 18 2 36 40 16610118

[B42] Smith DM Strain MC Frost SD Pillai SK Wong JK Wrin T Liu Y Petropolous CJ Daar ES Little SJ Lack of neutralizing antibody response to HIV-1 predisposes to superinfection Virology 2006 355 1 1 5 10.1016/j.virol.2006.08.009 16962152

[B43] Blish CA Dogan OC Derby NR Nguyen MA Chohan B Richardson BA Overbaugh J Human immunodeficiency virus type 1 superinfection occurs despite relatively robust neutralizing antibody responses J Virol 2008 82 24 12094 12103 10.1128/JVI.01730-08 18842728 PMC2593335

[B44] Gerhardt M Mloka D Tovanabutra S Sanders-Buell E Hoffmann O Maboko L Mmbando D Birx DL McCutchan FE Hoelscher M In-depth, longitudinal analysis of viral quasispecies from an individual triply infected with late-stage human immunodeficiency virus type 1, using a multiple PCR primer approach J Virol 2005 79 13 8249 8261 10.1128/JVI.79.13.8249-8261.2005 15956571 PMC1143736

[B45] Kozaczynska K Cornelissen M Reiss P Zorgdrager F van der Kuyl AC HIV-1 sequence evolution in vivo after superinfection with three viral strains Retrovirology 2007 4 59 10.1186/1742-4690-4-59 17716368 PMC2020475

[B46] Pernas M Casado C Fuentes R Perez-Elias MJ Lopez-Galindez C A dual superinfection and recombination within HIV-1 subtype B 12 years after primoinfection J Acquir Immune Defic Syndr 2006 42 1 12 18 10.1097/01.qai.0000214810.65292.73 16763489

[B47] Takehisa J Zekeng L Ido E Mboudjeka I Moriyama H Miura T Yamashita M Gurtler LG Hayami M Kaptue L Various types of HIV mixed infections in Cameroon Virology 1998 245 1 1 10 10.1006/viro.1998.9141 9614862

[B48] Ssemwanga D Lyagoba F Ndembi N Mayanja BN Larke N Wang S Baalwa J Williamson C Grosskurth H Kaleebu P Multiple HIV-1 infections with evidence of recombination in heterosexual partnerships in a low risk Rural Clinical Cohort in Uganda Virology 2011 411 1 113 131 10.1016/j.virol.2010.12.025 21239033 PMC3041926

[B49] Templeton AR Kramer MG Jarvis J Kowalski J Gange S Schneider MF Shao Q Zhang GW Yeh MF Tsai HL Multiple-infection and recombination in HIV-1 within a longitudinal cohort of women Retrovirology 2009 6 54 10.1186/1742-4690-6-54 19493346 PMC2700066

[B50] Herbinger KH Gerhardt M Piyasirisilp S Mloka D Arroyo MA Hoffmann O Maboko L Birx DL Mmbando D McCutchan FE Frequency of HIV type 1 dual infection and HIV diversity: analysis of low- and high-risk populations in Mbeya Region, Tanzania AIDS Res Hum Retroviruses 2006 22 7 599 606 10.1089/aid.2006.22.599 16831083

[B51] Yerly S Jost S Monnat M Telenti A Cavassini M Chave JP Kaiser L Burgisser P Perrin L HIV-1 co/super-infection in intravenous drug users AIDS 2004 18 10 1413 1421 10.1097/01.aids.0000131330.28762.0c 15199317

[B52] Grobler J Gray CM Rademeyer C Seoighe C Ramjee G Karim SA Morris L Williamson C Incidence of HIV-1 dual infection and its association with increased viral load set point in a cohort of HIV-1 subtype C-infected female sex workers J Infect Dis 2004 190 7 1355 1359 10.1086/423940 15346349

[B53] Redd AD Mullis CE Serwadda D Kong X Martens C Ricklefs SM Tobian AA Xiao C Grabowski MK Nalugoda F The Rates of HIV Superinfection and Primary HIV Incidence in a General Population in Rakai, Uganda J Infect Dis 2012 206 2 267 274 10.1093/infdis/jis325 22675216 PMC3415936

[B54] Cornelissen M Pasternak AO Grijsen ML Zorgdrager F Bakker M Blom P Prins JM Jurriaans S van der Kuyl AC HIV-1 dual infection is associated with faster CD4+ T-cell decline in a cohort of men with primary HIV infection Clin Infect Dis 2012 54 4 539 547 10.1093/cid/cir849 22157174

